# Supplementary figures and images for: Genome-scale model of metabolism and gene expression provides a multi-scale description of acid stress responses in Escherichia coli
Source: PLoS Comput Biol. 2019 Dec 6;15(12):e1007525. doi: 10.1371/journal.pcbi.1007525 (PMC6897400; doi:10.1371/journal.pcbi.1007525)

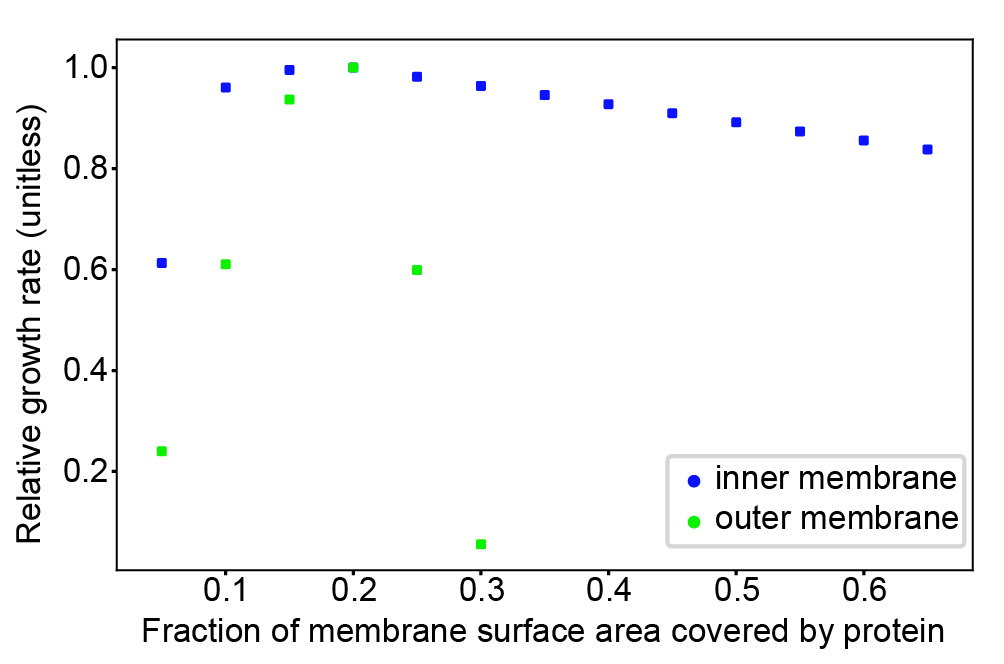

Supplement: S1 Fig — We examined the change in protein fraction in both the inner and outer membranes. Similar results in an earlier version of the ME-model can be found in the work by Liu et al [33] (Fig 5A). It is worth mentioning that the qualitative trend in terms of the change of growth rate matches with the earlier work, but discrepancies in the quantitative change of growth rate exist. Such discrepancies are mainly due to the change in membrane composition description in the latest version of the ME-model [27], which was used as the framework in this study. (TIF) [file pcbi.1007525.s001.tif]

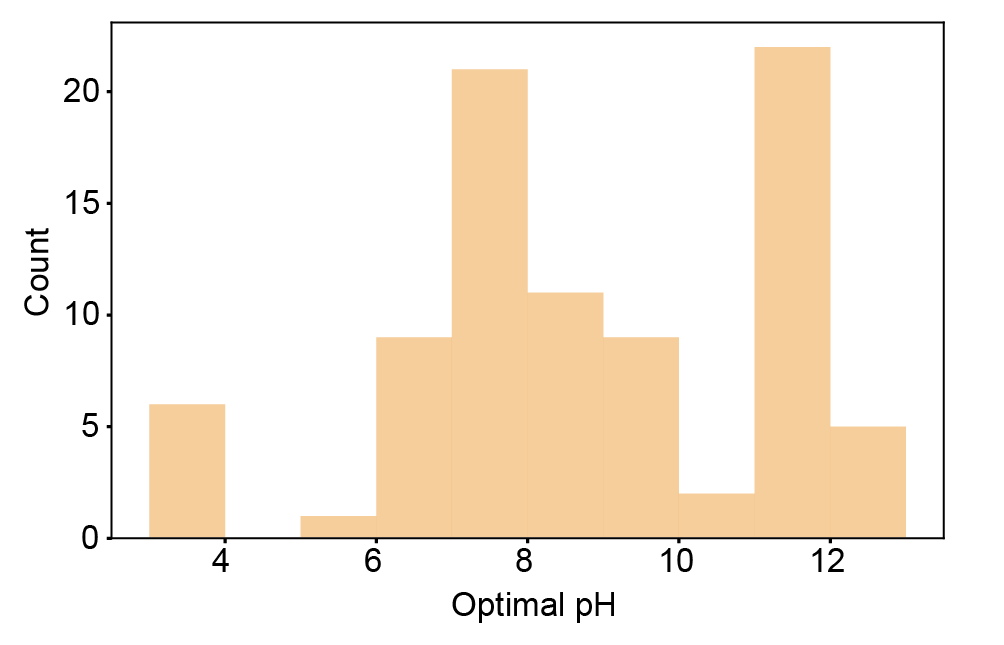

Supplement: S2 Fig — The optimal pH of the specific protein is determined based on the pH where the folding energy is the lowest. (TIF) [file pcbi.1007525.s002.tif]

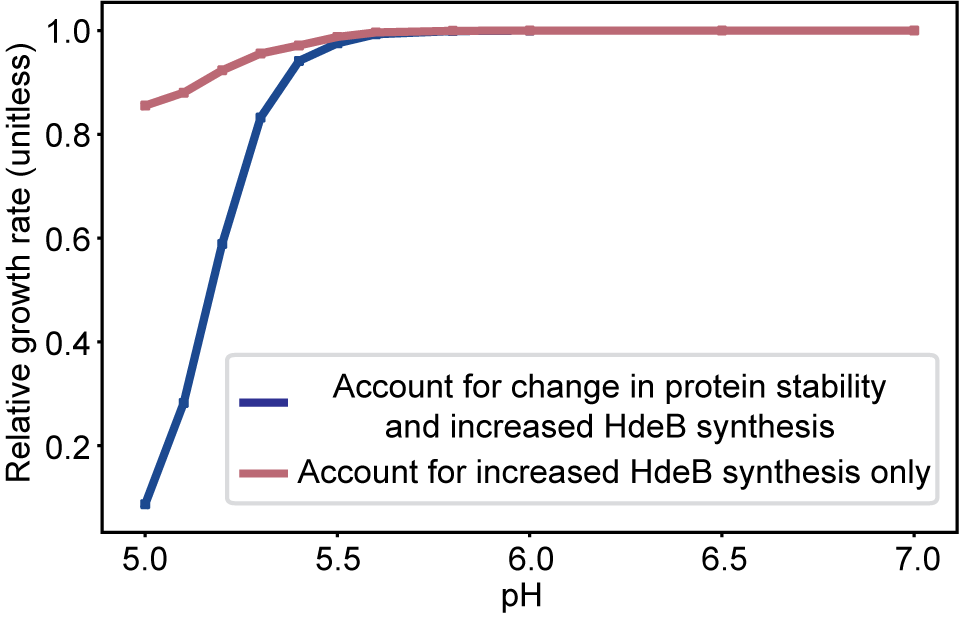

Supplement: S3 Fig — We found that when considering the the change in protein stability under acidic conditions, the growth rate dropped significantly, compared to when only considering the increased HdeB synthesis. (TIF) [file pcbi.1007525.s003.tif]

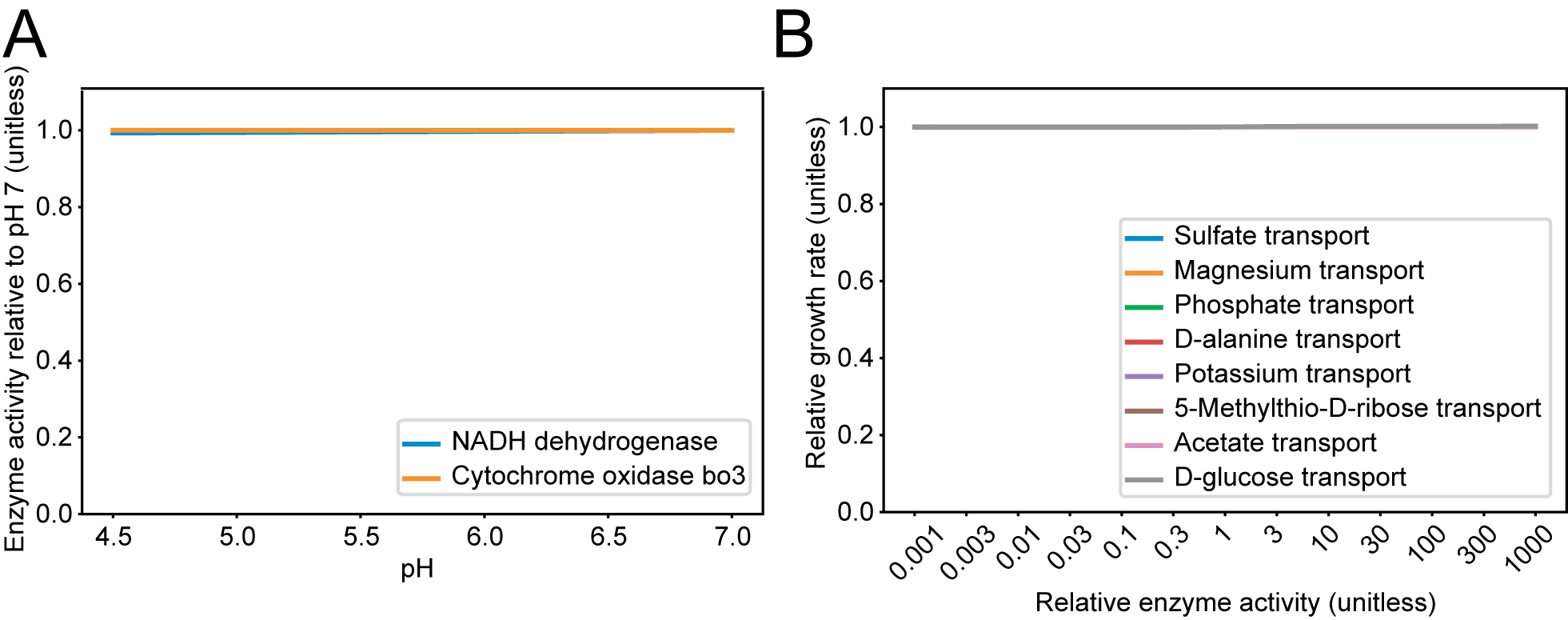

Supplement: S4 Fig — (A) Change in enzyme activity of electron transport chain (ETC) components as a function of pH. Here we focused on the ETC components active in the ME-model and calculated the change of their activity at different external pH values based on the theory of nonequilibrium thermodynamics (main text Materials and methods). We found that the two electron transport chain components examined does not have a notable change in enzyme activity across pH. (B) Change in growth rate due to change in the activities of membrane transporters. We focused on the membrane transporters that are active in the ME-model simulations. We change the activity of the membrane transporters one at a time and simulated the corresponding growth rates. We found that the change in the activities of membrane transporters do not significantly affect the growth rate (stayed at 1.0 relative growth rate). (TIF) [file pcbi.1007525.s004.tif]
