# Supplementary material for: Genome-scale model of metabolism and gene expression provides a multi-scale description of acid stress responses in Escherichia coli
Source: PLoS Comput Biol. 2019 Dec 6;15(12):e1007525. doi: 10.1371/journal.pcbi.1007525 (PMC6897400; doi:10.1371/journal.pcbi.1007525)
Supplement: Attachment — Submitted filename: Response to reviewers.pdf [file pcbi.1007525.s018.pdf]

Reviewer's Responses to Questions

Comments to the Authors:

Please note here if the review is uploaded as an attachment.

Reviewer #1: Based on an existing framework, this study characterizes the three acid stress mitigation responses of E. coli: 1) change in membrane lipid fatty acid composition, 2) change in periplasmic protein stability over external pH and periplasmic chaperone protection mechanisms, and 3) change in the activities of membrane proteins. The predictions by the ME model with the three integrated mechanisms demonstrated good agreement with RNA sequencing data of E. coli on gene expression changes under acid stress. It is valuable to mathematically reproduce genome-wide changes, but I have several comments.

1) It is hard to understand how the authors integrate chaperone protection mechanisms. How did they estimate the HdeB amount necessary for folding acid-induced unfolded proteins at different pHs? Did they estimate or measure the total protein amounts in periplasm? While Eq.4 does not show any rate equations, how did they estimate the synthesis flux of HdeB at different pHs?

We thank the reviewer for pointing this out and are happy to clarify here. First of all, the total amount of protein in the periplasm is computed by the ME-model subject to global constraints on the overall protein amount in the cell. The model is a continued development of the existing work [1, 2] and the constraints are the same as in the previously validated *E. coli* ME model framework, where the fraction of protein mass in different cellular compartments were verified by *in vivo* measurement. Next, we are unable to estimate the amount of HdeB in the periplasm at different pH conditions since there is no relevant data available. Instead, we let the model to produce just enough HdeB to bind and protect unfolded proteins in the periplasm. We did not use a rate equation here, since the ME-model framework is based on the steady state assumption, where the flux of HdeB needed is calculated based on mass balance. Specifically, we calculate the amount of unfolded proteins in the periplasm under different pH conditions, using equation 1 and 2 in the main text. Based on the amount of unfolded proteins, the flux of HdeB synthesis is computed by the model to meet the requirement of binding and protecting unfolded proteins in the periplasm. We have added the corresponding explanation in the Materials and Methods section at line 572 - 576.

1. Lloyd CJ, Ebrahim A, Yang L, King ZA, Catoiu E, O'Brien EJ, et al. COBRAme: A computational framework for genome-scale models of metabolism and gene expression. PLoS Comput Biol. 2018;14: e1006302
2. Liu JK, O'Brien EJ, Lerman JA, Zengler K, Palsson BO, Feist AM. Reconstruction and modeling protein translocation and compartmentalization in Escherichia coli at the genome-scale. BMC Syst Biol. 2014;8: 110.

2) More explanation on the ME model is required. Especially, how does it estimate macromolecule or chaperone synthesis flux? Does it consider transcriptional regulations (e.g. sigma factors)? Because readers like to know how chaperone synthesis regulation quantitatively affects the genome-scale metabolism.

Macromolecules are synthesized at the amounts needed to maintain the reaction flux catalyzed by the macromolecule, based on the mass balance constraint under the steady state assumption. Coupling coefficients are important terms linking the required amount of macromolecule synthesis flux and the flux of the catalyzed reaction. Coupling coefficients are generally expressed in the form of "μ/k_{eff}", taking into account the dilution rate of macromolecules into daughter cells with cell growth rate μ and the effective turnover rate (k_{eff}) of the process catalyzed by the macromolecule. Therefore, the flux of macromolecule synthesis can be expressed as $\mu/k_{eff} * v_{reaction}$, where $v_{reaction}$ is the flux of the catalyzed reaction. The reviewer can find more details in Figure 3 of the work by Lloyd et al [1].

The chaperone synthesis flux is determined based on the mass balance constraint. The amount of chaperones required is determined by the total amount of unfolded proteins in the periplasm. Based on equation 4 in the main text, we have $[HdeB] = \sum_{p=1}^{n}[Unfolded]_p\left(\frac{1+K_p+\mu_p/k_{folding-p}}{K_p}\right)$ that describes the amount of HdeB needed to bind the unfolded states of all n proteins in the periplasm. We have included this expression in line 576.

The sigma factor network is included in the latest ME-model framework [1]. However, we have not yet accounted for the transcription factor-based regulation in the framework. Therefore, we also do not account for transcriptional regulation of chaperone synthesis. However, the ME-model framework does compute the optimal synthesis rate of HdeB under varying pH conditions with the objective to maximize biomass production.

1. Lloyd CJ, Ebrahim A, Yang L, King ZA, Catoiu E, O'Brien EJ, et al. COBRAme: A computational framework for genome-scale models of metabolism and gene expression. PLoS Comput Biol. 2018;14: e1006302

3) Did the authors evaluate or measure decreased activities of membrane proteins (e.g. transporters)? Membrane protein activity would decrease because the periplasm sides of the proteins are exposed to a low pH.

We did consider the potential effect of acidic pH on the activities of membrane proteins. We focused the analysis within the scope of the ME-model framework and examined the membrane proteins that are active (carry nonzero flux) in the ME-model. We first evaluated the change in activity for ATP synthase and electron transport chain components from neutral pH to acidic pH. While the activity change of ATP synthase is significant and the effect was examined in detail in the main text (Figure 4), we found the rates of electron transport chain components to be almost unchanged from neutral pH to acidic pH (Figure S4A). For those membrane transporters that

are active in the ME-model, we performed a sensitivity analysis where we varied the activities of the transporters from 0.001 to 1000 fold and found that the change of their activities had minimal impact on cellular growth rate and processes (<1%) (Figure S4B).

4) The simulated cell growth decreased under acid stress. Is it caused by increased chaperone synthesis? Are there any other reasons for the decreased growth?

We found that decrease in simulated growth rate to be mainly caused by the change in fatty acid composition, periplasmic proteome and chaperone synthesis.

For the change in fatty acid composition, the increased fraction of cyclopropane fatty acids results in an increased demand of cofactor S-adenosyl-L-methionine (SAM). After donation of the methyl group, the cofactor goes through a series of steps to recover back to SAM. Specifically, SAM was first converted to S-adenosyl homocysteine after donating the methyl group. S-adenosyl homocysteine is hydrolyzed to homocysteine and adenosine by S-adenosylhomocysteine hydrolase. Homocysteine can then be converted to methionine through donation of a methyl group from 5-methyltetrahydrofolate catalyzed by methionine synthases. Methionine is then converted to SAM using ATP, as catalyzed by methionine adenosyltransferase. Thus the increased demand in SAM increases the use of ATP coupled to the process. As we compared the fluxes with and without such change, we found that increased demand in SAM results in decreased fluxes of ATP-consuming reactions, covering several key processes that will affect growth: ATP maintenance requirements, nucleotide metabolism (NDPK1, ADK1), glycolysis/gluconeogenesis (PFK, HEX1), etc. Reaction names are in bigg model notations (http://bigg.ucsd.edu) (The list of reactions and processes here are very similar to that of Table S5, which although is from a different context.)

Additionally, the change in periplasmic proteome also contributes to the decrease in growth rate. As we mentioned in the main text (line 248 - 251), one key protein that contributes to the decrease in growth rate is LptA protein, which is unstable under low pH. LptA protein is involved in the transport of (KDO)2-lipid IVA, which directly contributes to *E. coli* biomass [1]. Furthermore, we show that the increased chaperone synthesis also contributes to the decrease in growth rate. Specifically, in the ME-model, we gradually increase the flux in HdeB synthesis only and examined the change in growth rate (Figure S3 red line). The fluxes correspond to how much HdeB should be made when the model was under different pH conditions in the original simulations (Figure 3B). We found that increase in chaperone synthesis alone results in the decrease of growth rate in a less significant way, compared to the combined effect of changing protein stability and increased chaperone synthesis (Figure S3 blue line). This result indicates that the change of periplasmic protein stability is the main driver here. We have also included the discussion on factors that contribute to decreased growth rate under acidic conditions at line 247 to 250.

1. Sperandeo P, Cescutti R, Villa R, Di Benedetto C, Candia D, Dehò G, et al. Characterization of lptA and lptB, two essential genes implicated in lipopolysaccharide

transport to the outer membrane of Escherichia coli. J Bacteriol. 2007;189: 244–253.

5) In Fig 4A, the ATP synthesis rate was very high at pH 5. Can they explain how the produced ATP is used within a cell?

We have analyzed in detail the reactions that use ATP and list 50 reactions with the largest increase in ATP use (Table S5) when comparing acidic pH to neutral pH. The total change in ATP use of these 50 reactions account for ~94% of the additional ATP produced under acidic pH compared to neutral pH. We found that reactions with the largest increase in ATP use include ATP maintenance requirement, reaction catalyzed by phosphofructokinase, reaction catalyzed by nucleoside-diphosphate kinase, reaction catalyzed by adenylate kinase. The processes these reactions cover include nucleotide salvage pathway, glycolysis/gluconeogenesis, amino acid metabolism, purine and pyrimidine biosynthesis, translation process, etc. The results here also match with the results on the genes with the largest change in expression levels (Figure 4). We have also included the statement on reactions with the largest change in ATP use at line 313 to 316.

6) The ATP synthesis flux (Fig 4) is critically important in this study, thus it seems necessary to experimentally measure the ATP synthesis flux.

The ATP synthesis flux was estimated based on experimentally measured data, and we explained in line 589 - 593. Specifically, we obtained the rate of *E. coli* ATP synthase measured under three different transmembrane potentials (80 mV, 108 mV, 152 mV) and as a function of transmembrane pH difference [1]. We obtained a different set of kinetic parameters at each membrane potential by fitting the experimental data against equation 5 in the main text. To calculate ATP synthesis flux under the given external pH, we first calculated the cytoplasmic pH and the membrane potential and then used estimated parameter set with the closest membrane potential to plug into equation 5. It is worth mentioning that we used the effective turnover rate (k_{eff}) to describe the activity of the enzyme, which does not directly match up with the experimentally measured reaction rate. Therefore, we describe the change in activity through fold change at different pH conditions. A 5-fold increase in reaction rate correspond to a 5-fold increase in k_{eff}. We would also like to mention that the pH conditions used to calculate ATP synthesis flux are within the range of the pH conditions of the experimental data.

1. Fischer S, Gräber P. Comparison of DeltapH- and Delta***φ***-driven ATP synthesis catalyzed by the H(+)-ATPases from Escherichia coli or chloroplasts reconstituted into liposomes. FEBS Lett. 1999;457: 327–332.

7) In Fig 5B, “Others” are correctly predicted by the ME model. However, “others” are meaningless. Can they specify the gene names or their functions?

We agree that the “Others” term needs more details. Thus, we have expanded this category further into several COG processes, including amino acid metabolism, carbohydrate metabolism, cell Wall processes, cofactor metabolism, energy production and conversion,

intracellular trafficking, lipid metabolism, transcription. The changes are reflected in Figure 5B. Additionally, we have included the list of correctly predicted genes and their related processes in Table S6a-e for the five *E. coli* strains.

Reviewer #2: The authors extend their genome-scale model of *E. coli* metabolism to account for the effects of mildly acidic extracellular conditions. The model development is well explained and the comparisons to published data are promising. I have no major concerns about the study, but I do believe that the manuscript might be improved if the following comments are addressed:

1. The author summary reads too much like the abstract and should be rewritten from a broader perspective.

We thank the reviewer for pointing out this issue. We have rewritten summary based on the reviewer's suggestion, in line 37 - 51.

2. The five E. coli strains used should be mentioned earlier in the paper, like line 129.

We thank the reviewer for raising this point. The five *E. coli* strains mentioned here is actually different from those found in other parts of the text. The five strains at line 129 in the earlier version (now line 145) refer to the strains with membrane lipid fatty acid composition profiles measured in Brown et al [1]. For everywhere else in the text, the five strains refer to those with RNA sequencing data available grown under pH 5.5 and 7 [2]. For clarification, we have removed "five strains" in line 145 and mentioned the literature source right after.

1. Brown JL, Ross T, McMeekin TA, Nichols PD. Acid habituation of Escherichia coli and the potential role of cyclopropane fatty acids in low pH tolerance. Int J Food Microbiol. 1997;37: 163–173.
2. Du B, Olson CA, Sastry AV, Fang X, Phaneuf PV, Chen K, et al. Adaptive laboratory evolution of Escherichia coli under acid stress. Microbiology. 2019. https://doi.org/10.1099/mic.0.000867.

3. The authors should reconcile the statement on page 18 "We found the rates of electron transport chain components to be almost unchanged (<1%) from neutral pH to acidic pH. We were unable to calculate the reaction rates for most of the membrane transporters, due to missing metabolite concentration data. However, we found that the change of their activities had minimal impact on cellular growth rate and processes (<1%) through the sensitivity analysis using the ME-model" with the comment on page 20 "Additionally, we found upregulated expression for a number of proteins on the inner and outer membranes of E. coli. These proteins include the electron transport chain components,...".

We thank the reviewer for pointing out the inconsistent statements and would like to clarify further here. For the statement on page 18 (now line 264-265, 297, 300), we are discussing

specifically about the reaction rate of the electron transport chain components, or the rate at which the reactants are converted to products. On the other hand, on page 20 (now line 371), we are discussing the expression level of a number of electron transport chain components, or the amount of proteins that are produced. These two terms are not equivalent and the change of one does not necessarily affect the state of the other. For the context here, the main factor that influences the reaction rate is the external acidic pH, while a number of factors such as mass balance constraints on the metabolites involved and resource allocations can affect the expression level of a given protein.

4. Is there some reason that the DEGs were not analyzed for false negatives (i.e. the model predicts not change, but the genes are differentially expressed in experiment)? See lines 316-319.

We first include the relevant text here for direct reference: “We grouped the DEGs found in RNA-seq data into three categories: 1) DEGs currently not active in the ME-model, 2) DEGs correctly predicted by the ME-model, and 3) DEGs incorrectly predicted by the ME-model.”

We did not discuss the model predictions based on the confusion matrix here. Rather we choose a different way to categorize the model predictions. The categories listed here still cover the four different boxes of the confusion matrix, and we would like to clarify on the false negatives as the reviewer mentioned. Category 1 overlaps with part of the false positives, where the gene has an expression change between different conditions, but in the model the gene is not active (not carrying flux) due to the model limitations. These genes are listed in Table S6h and also briefly discussed in the text (line 337 - 343). Additionally, category 3 also overlaps with false positives, where the differentially expressed gene is active in the model, but predicted to have no change in expression by the model under different conditions. While such genes exist, the majority of genes in category 3 are those predicted by the model to have the wrong direction of change, including predicted upregulation but downregulation in data and predicted downregulation but upregulation in data, as discussed in detail in line 404 - 418.

5. The reason for limiting the modeled pH range from 5.5-7.0 should be mentioned earlier in the paper. See lines 416-431.

We thank the reviewer for the comment. We have reordered this text by moving it to the introduction (line 90 - 105). We make it clear why we focus on pH range 5.5 - 7.0 and mention why we do not include certain acid resistance mechanisms.

Reviewer #3: In the present manuscript, Du et al aim to get a systems-level understanding of the acid stress response of E. coli. Towards this end, the authors incorporate three known acid resistance mechanisms into a previously developed genome-scale metabolic and expression (ME) model of E. coli. In a first step, they consider these mechanisms (namely altered fatty acid

composition in membranes, periplasmic protein stabilization by chaperones, and changes in ATP synthase/ETC protein activity) separately and show that their model recapitulates previously reported behavior. For example, the authors predict that an acid-adapted fatty acid profile is accompanied by a reduction in growth rate, as is also observed experimentally. Finally, the authors combine all three acid resistance mechanisms to predict the response of their ME model to mild acid stress. As a metric of predictive power, the authors compare predicted and measured changes in gene expression, and show for 80% of the genes predicted to be upregulated in acid stress the experimental data shows a consistent upregulation.

Overall, the manuscript tackles an interesting biological problem, and presents the model implementation of different acid resistance mechanisms in a very clear fashion. However, several points in the final part of the manuscript were unclear to me:

1) The stated goal of this manuscript is to provide a “fundamental understanding” (lines 18-19) of the acid stress response. However, I felt that the manuscript struggles to provide an actual systems-level understanding of this response. For example, the authors report numerous expression changes in amino acid metabolism and translation genes, which are not obvious (at least to me). Can the authors provide a rationale for these changes? Are these a consequence of e.g. massive overexpression of HdeB (which according to the authors accounts for up to 25% of the protein mass at pH 5)? Currently, the manuscript relies too heavily on merely describing the (predicted or observed) changes in expression, without necessarily providing insights on why these changes might occur.

We agree with the reviewer that providing insights and rationale for gene expression change is necessary to gain a fundamental understanding of the acid stress response.

For the upregulated genes involved in the translation process in Figure 5, we found that the increase in ATP synthase activity to be the main contributor. We found significant overlap in the upregulated genes in the translation process when simulating with increased ATP synthase activity alone (Table S4) and with all acid stress response mechanisms added (Table S6f) (line 368 - 371). Increasing the activity of ATP synthase results in the upregulation of various ATP consuming reactions, as shown in Table S5 on reactions with the largest change in ATP use. Therefore, the increased demand in proteins to catalyze these reactions leads to the upregulation of genes involved in the translation processes, e.g. ribosome biosynthesis.

For the genes that change in amino acid metabolism as shown in Figure 5A, we examined the possible causes for such change from two aspects: 1) the metabolic processes that would require such changes, 2) the general processes that would require the proteome resources (as composed by these amino acids) under acid stress. First, we examined the downstream metabolic processes that would require the additional amino acid synthesized. Comparing the fluxes under acidic pH against those under neutral pH, we found that the increase in biosynthesis of methionine is one of the main processes consuming the increase in cysteine biosynthesis. Methionine is an important metabolite involved in the recycling of S-adenosyl-L-methionine (SAM), upon *cfa* upregulation in the adjustment of membrane lipid

fatty acid composition. Additionally, the increased biosynthesis of threonine mainly contributes to the biosynthesis of glutamate. The increased biosynthesis of glutamate contributes to several metabolic processes: 1) biosynthesis of lysine through aspartate (later for protein synthesis), 2) biosynthesis of asparagine (later for protein synthesis), 3) recycling of SAM (glutamate leads to methionine and tetrahydrofolate, which are intermediates of the SAM recycling process). Next, we examined the processes that have the largest change in proteome resource requirement under acid stress. For each amino acid in Figure 5A, we obtained the top 100 translation reactions in the ME-model that has the largest change in flux under acid stress, respectively. We then obtained the overlapping translation reactions from those amino acids. We examined the specific proteome sectors that correspond to the protein product of these translation reactions. We found that processes such as oxidative phosphorylation, cofactor and prosthetic group biosynthesis, glycolysis/gluconeogenesis to be the main categories requiring additional proteome resources (Table S7), and hence are the major drivers behind the upregulation of amino acid biosynthesis shown in Figure 5A.

Additionally, as mentioned in the main text at line 253 to 255 and line 378 - 381, the expression changes of genes involved in Sec translocon, lipid, glycerophospholipid and lipopolysaccharide metabolism are closely related to 1) the change in membrane lipid fatty acid composition, and 2) the stability change of the corresponding proteins (e.g. LptA) in the periplasm under acid stress, where the decreased amount of stable proteins in the periplasm increased the demand in the translocation of the stable proteins from cytoplasm. The expression changes of genes involved in cofactor and prosthetic group biosynthesis (e.g. folate and flavodoxin related genes) are mainly related to the recycling of cofactor S-adenosyl-L-methionine, due to the upregulation of *cfa* to adjust the membrane lipid fatty acid composition under acid stress (line 375 - 378).

2) It was unclear to me why the authors focus on upregulated genes (i.e. Figure 5B, lines 329-330). Given that the ME model includes proteome allocation, I would expect some parts of the proteome to shrink as well, right? In general, I would like to see a table with the actual model predictions and the experimental RNAseq data in the supplementary material of the final manuscript.

We have included Table S6a-e on differentially expressed genes (DEGs) found in RNA-seq data of the five *E. coli* strains. The list of DEGs between acidic pH and neutral pH is obtained based on the adjusted p-value of 0.05 and log2 fold change greater than 1. For the DEGs in each strain, we listed the log2 fold change in expression under acidic pH compared to neutral pH, as well as whether the direction of change is correctly or incorrectly predicted by the ME-model, or such gene is included but not active in the model, or not included in the model at all. We have also included Table S6f on the list of active genes in the ME-model and the predicted direction of change in expression under acidic pH compared to neutral pH. Lastly, we also have Table S2h on the list of genes not active in the ME-model but found to be differentially expressed in RNA-seq data, as also mentioned in the main text at line 337 - 343. Here, we attempt to validate the model predictions based on RNA-seq data. We found that for the downregulated genes in RNA-seq data, most of them are either inactive or not present in the

ME-model due to current model scope and very few are correctly or incorrectly predicted by the ME-model (Table S6a-e). Thus, we focus mainly on the upregulated genes in the RNA-seq data and the corresponding model predictions.

3) Ultimately, in its current form this manuscript is a well-executed consistency check: based on what we know about the molecular mechanisms of acid stress resistance, do we predict a cellular response to acid stress that is consistent with experimental data? But as the authors state themselves (lines 433-434), a key asset of such a model is its ability to design intervention strategies to reduce acid tolerance. However, the authors currently refrain from predicting intervention strategies (barring the short discussion in lines 434-438), which is a pity. I would suggest that the authors include a results section, in which they examine at least some potential intervention strategies, with a focus on those interventions that are also experimentally feasible (even if the authors decide not to test any of these interventions themselves).

We thank the reviewer for the comment and agree that demonstrating specific intervention strategies on acid tolerance using the ME-model can be helpful to highlight the model use cases. We thus have included a result section on two intervention strategies that reduce acid tolerance of *E. coli* (line 432 to 463). Based on the acid stress responses included in the ME-model, these two strategies are downregulation of HdeB protein and knockout of the *cfa* gene.

We first showed the effect of HdeB downregulation on the model under pH 5.5. We would like to mention that since we have HdeB as the only chaperone protecting the periplasmic proteome under acidic conditions, knockout of the *hdeB* gene would result in no growth in ME-model simulations. Thus, we simulated the ME-model with 10% of the original HdeB protein amount under pH 5.5. We found the growth rate to drop to 31.6% of the original value under pH 5.5. We found that genes with the largest change in expression level include *ilvBHIN* (amino acid metabolism), *lysS* (tRNA charging), *rpe* (pentose phosphate pathway), *lysU*, *deoA*, *udp*, *tdk*, *yjjG* (nucleotide salvage pathway) (Table S9). This intervention can be a good case study for experimental validation, by knocking out the *hdeB* gene and analyzing with RNA sequencing data. If there are discrepancies between the model simulation and the data, this can potentially lead to the discovery of novel chaperone protection mechanisms in the periplasm.

Next, we model the effect of *cfa* gene knockout under acidic conditions. This change affects the reactions catalyzed by cyclopropane fatty acyl phospholipid synthase (CFAS), which converts unsaturated fatty acids to cyclopropane fatty acids. As a result of *cfa* knockout, *E. coli* will be unable to synthesize cyclopropane fatty acids under acidic conditions. Thus, the membrane fluidity of *E. coli* is likely to be similar to that under neutral condition, while increased proton gradient under acidic conditions increase the leakage of protons into the cytoplasm [1-3]. Thus, besides deactivating reactions catalyzed by CFAS, we also model the effect of proton leakage into the cytoplasm. We show that with *cfa* knockout and increased proton leakage, genes with the largest change in expression cover processes including amino acid metabolism (*glnA*, *argBC*), carbohydrate metabolism (*paaH*, *idnK*), oxidative phosphorylation (*atpADE*), membrane

and transport related processes (*lpp*, *ompN*, *hcaT*) (Table S10). Again, any discrepancies between the model simulation and the data might suggest novel strategies that *E. coli* uses to adjust membrane lipid fatty acid composition.

1. Brown JL, Ross T, McMeekin TA, Nichols PD. Acid habituation of Escherichia coli and the potential role of cyclopropane fatty acids in low pH tolerance. Int J Food Microbiol. 1997;37: 163–173.
2. Chang YY, Cronan JE Jr. Membrane cyclopropane fatty acid content is a major factor in acid resistance of Escherichia coli. Mol Microbiol. 1999;33: 249–259.
3. Yuk H-G, Marshall DL. Adaptation of Escherichia coli O157:H7 to pH alters membrane lipid composition, verotoxin secretion, and resistance to simulated gastric fluid acid. Appl Environ Microbiol. 2004;70: 3500–3505.

Additional points:

1) Figure 1S: it was unclear to me what exact earlier results are being reproduced in this figure. Maybe add some more explanation in the figure caption, or include these previous results in the figure?

We thank the reviewer for pointing this issue out. We have included a specific reference to earlier results and provided some explanations in the figure caption for Figure S1.

2) I was intrigued by the model predicting that a change in FA composition is enough to reduce growth rate by ~10% (Figure 2B). Do the authors have an explanation for this? Along the same lines, the authors predict that Cfa has the largest change in expression in the acid-adapted profile. Would overexpression of Cfa be sufficient to a) change lipid composition and b) reduce growth rate?

We found decreased growth rate to be mainly associated with increased demand in ATP related to the recycling of S-adenosyl-L-methionine (SAM), which is an important cofactor to meet the requirement of fatty acid composition adjustment under acidic pH. Specifically, the SAM cofactor donates the methyl group to make cyclopropane fatty acid and becomes S-adenosyl homocysteine, which is then hydrolyzed to homocysteine and adenosine by S-adenosylhomocysteine hydrolase. Homocysteine can then be converted to methionine through donation of a methyl group from 5-methyltetrahydrofolate catalyzed by methionine synthases. Methionine is then converted to SAM using ATP, as catalyzed by methionine adenosyltransferase. Thus the increased demand in SAM increases the use of ATP coupled to this process. In fact, we found that increased demand in SAM results in decreased fluxes of several ATP-consuming reactions, covering several key processes that will affect growth: ATP maintenance requirements, nucleotide metabolism (NDPK1, ADK1), glycolysis/gluconeogenesis (PFK, HEX1), etc. Reaction names are in bigg model notations (http://bigg.ucsd.edu) (The list of reactions and processes here are very similar to that of Table S5, which although is from a different context.)

In the ME-model simulation, we enforced increased fluxes in reactions catalyzed by cyclopropane fatty acyl phospholipid synthase (the product of the *cfa* gene). The reviewer is right that we did observe decreased growth rate and change in membrane lipid fatty acid compositions, with a significant increase in the fraction of cyclopropane fatty acids. This shows the association between the overexpression of *cfa* gene and the change in membrane lipid fatty acid composition. In our ME-model framework, we impose the constraint directly on membrane lipid fatty acid composition and observe the genes and reactions contributing to such change. While the model is limited at identifying what causes the overexpression of the *cfa* gene, it is worth investigating the related upstream processes and triggers experimentally.

3) In lines 155 to 162 the authors refer to expression changes in various genes, without providing a pointer to a supplementary table of figure. Please make sure to include such a pointer. The same accounts for the text in lines 231 to 242.

We have included supplementary tables on the top genes with the largest change in expression. Table S1 lists the genes with the largest change under the adjustment of membrane lipid fatty acid composition (original line 155 - 162, now 172 - 179). Table S3 lists the genes with the largest change considering the change in periplasmic protein stability and periplasmic chaperone protection mechanisms (original line 231 - 242, now line 242 - 255).

4) Lines 201-202: "…indicating that protein stability might be an underlying factor that influences the protein expression in the periplasm." This sentence was unclear to me: do the authors suggest that designated regulatory mechanisms that expression the respective proteins in a pH-dependent manner?

The statement here is a hypothesis as we observed certain proteins to have a higher expression level [1] under their calculated optimal pH than under neutral pH. There can be different factors contributing to such higher expression level and protein stability might be one of them. To avoid confusion, we have removed this hypothetical statement.

1. Maurer LM, Yohannes E, Bondurant SS, Radmacher M, Slonczewski JL. pH regulates genes for flagellar motility, catabolism, and oxidative stress in Escherichia coli K-12. J Bacteriol. 2005;187: 304–319.

5) The authors identify LptA as "the major factor causing the drop in growth rate and increase in HdeB mass fraction" (lines 236-237). Do the authors predict that overexpression of LptA, or expression of an LptA protein variant from a related bacterial species with better lower pH tolerance, would alleviate the growth defect? If yes, are data on such a validation experiment available in the literature?

The LptA protein as the main factor causing the drop in growth rate is identified from ME-model simulations and analysis on its folding equilibrium. We did not observe a recovery in growth rate when overexpressing LptA in our model simulations. The main reason is that the decrease in LptA protein stability under acidic conditions prevents the presence of active LptA protein in the

periplasm. Since the equilibrium between the folded and unfolded states of the LptA protein strongly favors the unfolded state, any newly synthesized LptA protein transported into the periplasmic space will be in the unfolded state under acidic conditions. We redid the simulation by eliminating the change in stability of LptA protein across conditions and found the results to be similar to Figure S3, where the increase in chaperone synthesis mainly contributes to the decrease in growth rate. We did not find direct literature evidence of LptA protein variant in other bacterial species (based on MetaCyc) nor validation experiments on how LptA overexpression can alleviate the acid stress. However, the reviewer brings up a good point in terms of potential strategies to improve acid tolerance, that is looking for LptA variants with greater stability under acidic conditions. This point can also be used for future experimental validation with the expression of *lptA* gene variant in *E. coli* under acid stress.

6) To validate their predictions, the authors compare gene expression with RNA-seq data. I'm curious whether the authors can also use published proteomics data (i.e. from a data set in which E.coli was subjected to pH 6, PMID 26641532) for validation?

We have included the comparison of model prediction against the proteomics data by Schmidt et al [1], where *E. coli* was grown under pH 6 and pH 7. The results can be found in Table S8. Based on the distribution of confidence score of the proteins in data, we selected proteins with confidence score greater than 500. Comparing the direction of change in protein expression under acid stress from data and predicted by the model, we found that the expression change of 123 proteins were correctly predicted and 119 proteins were incorrectly predicted in data. It is interesting to note that the largest category for both the correctly and incorrectly predicted proteins is involved in the translation process. The model was able to correctly predict most of the upregulated genes in the translation process, but very few downregulated genes involved in the translation process. This result shows that by incorporating the different acid response mechanisms, the model is able to capture certain processes that become more active under acid stress (line 420 - 430).

1. Schmidt A, Kochanowski K, Vedelaar S, Ahrné E, Volkmer B, Callipo L, et al. The quantitative and condition-dependent Escherichia coli proteome. Nat Biotechnol. 2016;34: 104–110

7) Can the authors include the numbers of DEGs for the different categories mentioned in lines 316 – 318?

We have included the numbers in Table S6g. The numbers are obtained from the categories of model predictions on DEGs in RNA-seq data of five *E. coli* strains in Table S6a-e.

8) The authors should include the full list of DEGs in their final model (basis for Figure 5) in the supplementary material.

As in responses to reviewer 3's major point 2, we have included Table S6a-e on the DEGs found in RNA-seq data of the five *E. coli* strains. We have also included table S6f on the list of DEGs predicted by the ME-model.

Have all data underlying the figures and results presented in the manuscript been provided?

Large-scale datasets should be made available via a public repository as described in the *PLOS Computational Biology* data availability policy, and numerical data that underlies graphs or summary statistics should be provided in spreadsheet form as supporting information.

Reviewer #1: Yes

Reviewer #2: Yes

Reviewer #3: No: As described above, there are several instances in which the authors highlight example results, without providing a pointer to the full results/analysis.
